# A Microfluidic Chip Embracing a Nanofiber Scaffold for 3D Cell Culture and Real-Time Monitoring

**DOI:** 10.3390/nano9040588

**Published:** 2019-04-10

**Authors:** Jeong Hwa Kim, Ju Young Park, Songwan Jin, Sik Yoon, Jong-Young Kwak, Young Hun Jeong

**Affiliations:** 1 Department of Mechanical Engineering, Graduate School, Kyungpook National University, Daegu 41566, Korea; qhfekrn89@gmail.com; 2 Department of Mechanical Engineering, Pohang University of Science and Technology (POSTECH), Pohang 37673, Korea; juyoung1489@postech.ac.kr; 3 Department of Mechanical Engineering, Korea Polytechnic University, Siheung 15073, Korea; songwan@kpu.ac.kr; 4 Department of Anatomy, Pusan National University School of Medicine, Yangsan 50612, Korea; sikyoon@pusan.ac.kr; 5 Department of Pharmacology, Ajou University School of Medicine, Suwon 16499, Korea; jykwak@ajou.ac.kr; 6 School of Mechanical Engineering, Kyungpook National University, Daegu 41566, Korea

**Keywords:** nanofibers, microfluidic chip, electrospinning, live assay, hepatocellular carcinoma cells

## Abstract

Recently, three-dimensional (3D) cell culture and tissue-on-a-chip application have attracted attention because of increasing demand from the industries and their potential to replace conventional two-dimensional culture and animal tests. As a result, numerous studies on 3D in-vitro cell culture and microfluidic chip have been conducted. In this study, a microfluidic chip embracing a nanofiber scaffold is presented. A electrospun nanofiber scaffold can provide 3D cell culture conditions to a microfluidic chip environment, and its perfusion method in the chip can allow real-time monitoring of cell status based on the conditioned culture medium. To justify the applicability of the developed chip to 3D cell culture and real-time monitoring, HepG2 cells were cultured in the chip for 14 days. Results demonstrated that the cells were successfully cultured with 3D culture-specific-morphology in the chip, and their albumin and alpha-fetoprotein production was monitored in real-time for 14 days.

## 1. Introduction

Over the last century, two-dimensional (2D) in-vitro cell culture has been used in studying the responses to stimulation from biological and biochemical materials, such as drugs, toxic materials, and detoxification. Traditional in-vitro cell tests are based on 2D culture on a flat surface, and the 2D culture environment is completely different from the human body. Cells inside the human body are surrounded by extracellular matrix (ECM) and tissue fluid in a complex three-dimensional (3D) space. Thus, cells are difficult to activate in 2D environments to maintain differentiation and expression of tissue-specific physiological functions and physiological activity [1,2].

Tissue-on-a-chip is a recapitulation of the biological and mechanochemical environment of human body tissues using a small device chip [1]. This technique includes cells, chemical and physical environments, and the microenvironment. It is used in the development of in-vitro disease models, drug screening, toxicity testing, and disease research, by providing a cellular environment that better mimics human physiological conditions [3,4,5]. Typical tissue-on-a-chip consists of an integrated microscale engineering system, several types of cells, and culture medium. The microelectromechanical system (MEMS) allows the use of microfluidic devices to reconstitute distinct features of the tissue–tissue interface, physiological movements, and biochemical environment similar to the human body. Marino et al. [6] presented a microfluidic system to mimic the blood–brain barrier (BBB). The system consisted of porous microtubes fabricated using two-photon lithography and enabled co-culturing brain microcapillary cells and functioning.

Recently, various 3D environmental features, such as microporous membrane [7,8], hydrogel [9,10,11], and nanofibers [12,13], have been introduced to tissue-on-a-chip applications to provide the similar structures and functions of the human body to cell culture. The hydrogel is highly permeable and an excellent biocompatible material. Annabi et al. coated microfluidic channels with synthesized photo-crosslinkable gelatin and tropoelastin-based hydrogel to improve cardiomyocyte culture in a polydimethylsiloxane (PDMS) surface [9]. Au et al. fabricated hepatic tissue models by encapsulating HepG2 and NIH-3T3 cells in a hydrogel. Their platform showed better results than 2D cell culture systems in drug screening [14]. Gumuscu et al. [15] proposed a microfluidic cell culture platform composed of 3D collagen hydrogel compartments, and they applied their system for co-culture of human intestinal cells and drug screening in preliminary level. Porous membranes are considered mimic basal membranes of barrier tissue, such as tissue–tissue, tissue–liquid, and tissue–air interfaces in organ-on-a-chip [16,17]. 

Nanofibers have diameters ranging from tens to hundreds of nanometers and similar morphology to the extracellular matrix of the human body. In particular, nanofibers are well suited for cell nutrient exchange, communication, and efficient cellular responses because of large surface areas, high porosity, and spatial interconnectivity [18,19]. As a result, nanofibers have been extensively applied to a variety of applications such as porous membrane and scaffold in biotechnology [20,21,22]. In addition, cells are easily attached and better proliferated in electrospun composite nanofiber than in a conventional 2D culture environment, such as a petri dish [23].

Here, we present a microfluidic chip with a nanofiber scaffold, which can provide a 3D human body ECM-like environment to cell culture and monitor cell status and activity using a conditioned culture medium. The nanofiber scaffold was electrospun so that it composed various diameter fibers, thereby providing highly porous morphology to cells under the well-defined microfluidic chip conditions. In particular, a perfusion method, which enables real-time monitoring cell status, was demonstrated. The developed chip was applied to 3D culture of HepG2 cells, which has various functions, such as cell growth and secretion of proteins. Our results demonstrated that HepG2 cells were cultured with 3D culture-specific morphology (i.e., large spheroids) [3,24], and their protein production was successfully monitored for 14 days.

## 2. Materials and Methods

### 2.1. Concept of Microfluidic Chip with Nanofiber Mat

**Figure 1:**
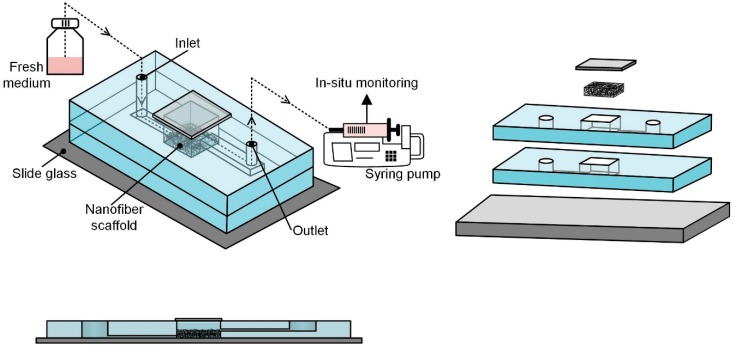
Concept of a microfluidic chip with a nanofiber scaffold.

Figure 1 shows the schematic concept of the proposed microfluidic chip embracing nanofibers, which consists of a nanofiber scaffold, a microfluidic chip structure, perfusion environment, and cells. The chip was designed to mimic the dynamic microenvironment of the human body, support perfusion-based long-term culture, and allow real-time monitoring of secretions and functionalities of the cultured cells in vitro. An electrospun nanofiber scaffold was introduced to a microfluidic chip to provide a 3D extracellular matrix (ECM)-like environment, because the nanofibers have similar morphology to the human body’s ECM [25]. The nanofiber scaffold was located on the chamber bottom. The microfluidic chip has a simple structure composed of micro-channels, a chamber, and gate holes, and it is made of Polydimethylsiloxane (PDMS). The cell suspension was introduced into the top opening of the chamber before closing the opening with a cover slip. Fresh culture medium was supplied to the cell-seeded nanofiber scaffold via the inlet microchannel to provide supplied oxygen and nutrients to the cells. The cell culture-conditioned medium through the outlet microchannel was collected to monitor cell activity. Fluidic connections to the microfluidic chip were made with tubing inserted through the inlet and outlet holes. Culture medium perfusion/flow in the chip was established with the help of a perfusion environment consisting of a fresh media reservoir at the side of the inlet and a syringe pump at the side of the outlet. Therefore, medium flow was derived by negative pressure. The collected conditioned medium in a syringe was easily transferred for analysis, such as enzyme-linked immunosorbent assay (ELISA).

As shown in Figure 1, the chip is composed of four layers: a supportive plate (slide glass), two microfluidics layers (PDMS), and a window to chamber. The microfluidic layer has a microchannel on the bottom plane and a rectangular hole as a chamber at the center of the layer. A thin coverslip was used as the window to chamber in this study. After assembling all the layers, the rectangular holes formed the center chamber with the supportive plate and window to chamber.

Flow rate of the culture medium through the chip was determined by considering the required amount of culture medium for cell growth, 2D culture condition, and allowable shear stress on the cells induced by culture medium flow in microfluidics. Information on the required amount of culture medium can be obtained from the culture product company, whereas the shear stress limit can be obtained from a previous study [26]. The amount of media supplement per hour (flow rate) can be determined from the following equation:

$$\frac{M}{N_{R}\times T}\times N_{chip}\leq \;q\;\leq \;\frac{\tau_{max}bh^{2}}{6\mu }$$

where *M* is the amount (volume) of culture medium supply for the number (*M_R_*) of cells at a given culture area. *T* is the medium exchange period. *M*, *N_R_*, and *T* are the recommended values under 2D culture condition. *N_chip_* is the number of cells seeded in the chip, *τ_max_* is the allowable shear stress loaded to cells to avoid cellular damage, *μ* is the viscosity of medium (g/cm∙s), *q* is the flow rate through the chip (cm^3^/s), and *b* and *h* are the width (cm) and height (cm) of the microchannel, respectively. The maximum limit of the flow rate, which was calculated from the allowable shear stress, was 1.54 mL/h. The minimum flow rate, which was calculated from the 2D culture protocol [27] considering the number of seeded cells in a scaffold and scaffold area, was 0.001 mL/h. We set the flow rate at 0.1 mL/h between the maximum and minimum limits.

### 2.2. Fabrication of Microfluidic Chip Structure

The microfluidic chip structure was made of PDMS using well-established soft lithography and micromolding [28,29] at a preliminary level. The master molds were prepared by cutting adhesive tape (3M) according to the channel shape, and removing the tape from all areas except the channel shape on a slide glass (ThermoFisher Scientific, Waltham, MA, USA)). PDMS (Dow Corning, Midland, MI, USA) was cast on the master molds using the pre-polymer (base) to curing agent weight ratio of 10:1, and the molds were cured in a dry oven at 60 °C for 3 h.

**Figure 2:**
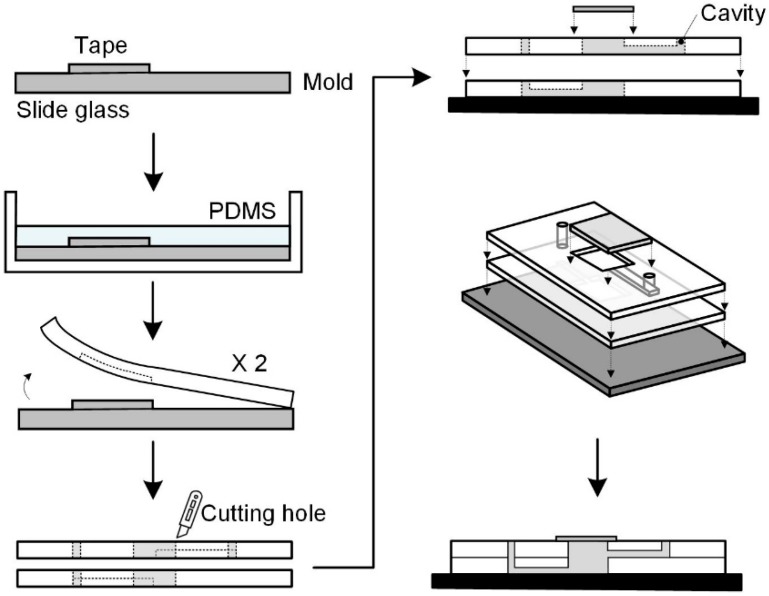
Fabrication of patterned microchannels using soft lithography.

In the microfluidic layer, the microchannels had a cross-section of about 300 × 100 μm and a length of about 10 mm, whereas the chamber hole had a rectangular shape with a size of about 12 × 12 mm and a height of about 3 mm. The microchannels were patterned using soft lithography (Figure 2). After curing, a rectangular chamber hole was made by cutting a rectangle in the cured PDMS slab. A hole to the inlet microchannel was made by using a biopsy puncher.

All the PDMS structures and slide glass were autoclaved for sterilization and bonded together after corona treatment (Electro-Technic Products Inc., Chicago, IL, USA) to secure tight bonding (Figure 1). Before inserting the nanofiber scaffold into the chamber, the scaffold was sterilized in 70% ethanol and thoroughly washed three times with PBS. A nanofiber scaffold was inserted into the chamber inside the bonded PDMS. The assembled chip samples were stored on a clean bench for a while. After seeding the cell suspension onto the nanofiber scaffold, the chamber was covered with glass coverslips. The microfluidic device was immobilized with two polycarbonate (PC) plates and screws to ensure no leakage.

### 2.3. Fabrication of Nanofiber Scaffold

In this study, 8 wt% polycaprolactone (PCL) solution was prepared by dissolving PCL with an average molecular weight (Mw) of 70,000–90,000 (PCL, Sigma-Aldrich, St.Louis, MO, USA) in 99.5% chloroform (Samchun Pure Chemical Co., Seoul, Republic of Korea). The mixture was then homogenized with a magnetic stirrer for 12 h at room temperature.

The electrospinning setup consisted of a high-voltage power supply, syringe pump, nozzle spinneret with an inner diameter of 210 μm, and a grounded solid drum collector, which rotated at a speed of about 30 rpm. The nozzle spinneret tip-to-collector distance was maintained at 100.0 mm. The electrical potential between the nozzle spinneret and the drum collector was 10.0 kV. The flow rate of the PCL solution was controlled by a syringe pump at 0.1 mL/h. The thickness uniformity of the nanofibers was improved by providing the nozzle spinneret with a traveling motion speed of 1 mm/s along the drum axis direction. The nanofibers were electrospun until they reached a thickness of 100 μm (actually, 113.7 ± 2.7 μm), which was measured using an ultra-precision micrometer (Mitutoyo) with a constant compression of 0.5 *N*. During electrospinning, the temperature and relative humidity were maintained at 19–20 °C and 50−55%, respectively. The electrospun nanofibers were provided with 5 min of heat treatment at 60 °C using a dry oven to improve nanofiber interconnectivity. The nanofibers were then cut into the same size of the chamber (12 × 12 mm) with a scalpel and carefully placed into the microfluidic chamber using a tweezer. The morphology of the electrospun nanofibers was observed by scanning electron microscopy (SEM; Hitachi, Tokyo, Japan). The porosity of the nanofibers was measured using a mercury porosimeter (Micromeritics Instrument Co., Norcross, GA, USA). The filling pressure of mercury was 1.23 psi, and the equilibrium time was 10 s.

### 2.4. Cell Culture and Flow Experiments

Human liver hepatocellular carcinoma cells (HepG2) were obtained from Korean Cell Line Bank. HepG2 cells in suspension containing 1 × 10^4^ cell/chamber were seeded on the nanofiber scaffold in the microfluidic chip under static conditions, and allowed to form a stable attachment for 24 h. After the cells were allowed to settle, the medium was continuously perfused to the chip at a flow rate of 0.1 mL/h using a commercial syringe pump (New Era Pump Systems, Farmingdale, NY, USA). The perfused culture medium was Dulbecco’s modified Eagle’s medium (Life Technologies, ThermoFisher Scientific, Waltham, MA, USA) supplemented with 10% fetal bovine serum (Life Technologies, ThermoFisher Scientific, Waltham, MA, USA) and 1% penicillin streptomycin. The cultures were stored for 14 days in a humidified atmosphere of 5% CO_2_ in air at 37 °C. We could not find any deterioration in the device operation nor change in the nanofiber morphology during the culture. Moreover, the nanofibers stuck tightly in the chamber and embedded well on the chamber bottom.

### 2.5. Functional Assays

To verify hepatocyte viability of the developed microfluidic chip, the cells on the scaffold after settling down were stained with Calcein-AM to indicate live cells and ethidium homodimer-1 to indicate dead cells, according to the manufacturer’s instructions (Life Technologies, ThermoFisher Scientific, Waltham, MA, USA). The live and dead cells were observed using a confocal microscope (Carl Zeiss, Oberkochen, Germany). We investigated cell proliferation behavior under static and perfusion conditions to justify the effect of medium perfusion with microfluidic chip. Unlike cell culture under perfusion condition, the medium in the chamber was exchanged with fresh medium daily by using a pipet to compare cell proliferation under both conditions. Cell proliferation was assessed by measuring the DNA content of the cultured cells in each chip. The DNA content was measured on days 1 and 14. DNA from cultured cells was extracted using a commercial kit (Qiagen, Hilden, Germany). The DNA contents for both static and perfusion conditions were quantified by spectrophotometers (Thermo Fisher Scientific, Waltham, MA, USA).

Here, we quantified the secretion of albumin and alpha-fetoprotein (AFP) using enzyme-linked immunosorbent assay (ELISA) to demonstrate the applicability of the developed microfluidic chip to the 3D cell culture and real-time monitoring with HepG2 cells. Therefore, the conditioned culture medium was continuously collected at a rate of 0.1 mL/h and harvested daily by changing the syringe in the syringe pump. The amount of albumin secreted from the HepG2 cells was quantified every 2 days until day 14 using a human albumin ELISA kit (Bethyl Laboratories, Montgomery, TX, USA). AFP secretion was measured using an AFP ELISA kit (CUSABIO, Wuhan, China) every 3 days, from day 9 to day 14, according to the manufacturer’s instructions. The required volume per sample of the conditioned medium for the assays was 100 μL, and the volume of the daily collected medium (2.4 mL) was sufficient to analyze various secreted proteins, up to 12 types, after duplication.

The morphology of the cells cultured in the microfluidic chip was investigated by SEM and fluorescent imaging. Cells cultured in the chips for 1 and 14 days were harvested with phosphate buffered saline (PBS) and then fixed with 4% paraformaldehyde solution. The samples for SEM were dried in a freeze-dryer (Labconco, Kansas City, MO, USA). Sample morphology was observed using SEM (Hitachi Hitachi, Tokyo, Japan). The cell morphology in the chip was assessed by staining F-actin with the phalloidin-fluorescein isothiocyanate (Phalloidin-FITC, Sigma-Aldrich, St. Louis, MO, USA) and nuclei with DAPI (4′,6-diamidino-2-phenylindole, Life Technologies, ThermoFisher Scientific, Waltham, MA, USA). The cells were then visualized using a confocal laser scanning microscope (Carl Zeiss, Oberkochen, Germany).

### 2.6. Statistical Analysis

All experiments were carried out with 5 samples (N = 5) except DNA content assay with four samples. All the samples were duplicated in assays. The experimental data are presented as means ± standard deviation (SD). ANOVA was conducted when we compared more than two groups for statistical comparison. The differences between the mean values of each group were evaluated by Student’s *t*-test, in which a *p* value less than 0.001 was considered significant.

## 3. Results and Discussion

### 3.1. Microfluidic Chip Embracing a Nanofiber Scaffold

**Figure 3:**
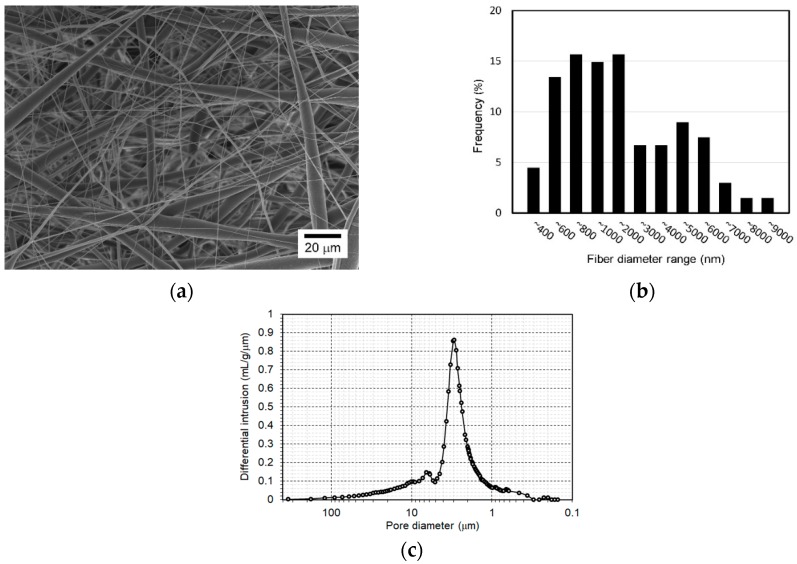
Electrospun nanofiber scaffold: (**a**) SEM images; (**b**) diameter distribution of the electrospun fibers; (**c**) porosity analysis result.

**Figure 4:**
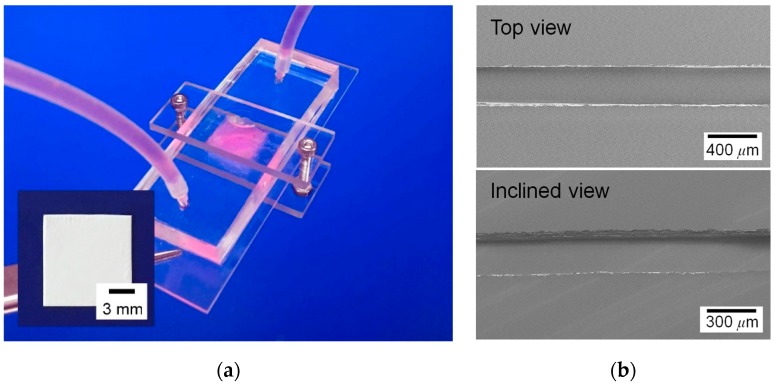
Microfluidic chip with a nanofiber scaffold (**a**) and SEM images of the microchannel of the chip (**b**).

Figure 3a shows the SEM image of the electrospun nanofiber scaffold for the proposed chip. First, the electrospun fibers were randomly oriented. Moreover, the nanofiber scaffold in this study had various diameter fibers due to the combination effect of the chloroform solvent, with faster evaporation, and the process conditions [30,31]. Some fibers had diameters ranging from several to 10 μm, whereas others appeared fine with diameters in the nanometer scale. Results of detailed analysis of fiber diameter distribution are in Figure 3b. The peak frequency diameter of fine fibers (i.e., nanofibers) was between 600 and 1000 nm, and a significant number of microfibers had a diameter of 2 to 6 μm in the scaffold. This nanofiber scaffold possibly contained larger pores than the scaffold consisting of small diameter nanofibers, because the microfibers can introduce larger pores than nanofibers [32]. Figure 3c shows the differential intrusion curve with respect to pore size of the nanofiber scaffold. From the figure, the pore diameter (equivalently, size) ranged between 0.3 and 100 μm and the peak frequency pore diameter was about 3 μm. The porosity of the nanofiber scaffold was about 76%. Therefore, the fabricated nanofiber scaffold may allow the cells to infiltrate into the deep regions of the scaffold. Figure 4a shows an assembled microfluidic chip embracing a nanofiber scaffold. The developed chip looks simple and is appropriate for use in a real PDMS fabrication environment. The microchannel of the chip had a width of 307.6 ± 4.5 μm and depth of 97.2 ± 4.9 μm, as shown in Figure 4b. The microchannel had a flat and smooth bottom plane, while its side walls had a rough surface because of the cut edge of tapes. However, the rough wall surface of the microchannel did not affect the device operation quality because the microchannel engaged only in the delivery of culture medium with slow velocity.

### 3.2. Application to 3D Cell Culture and Real-Time Monitoring

**Figure 5:**
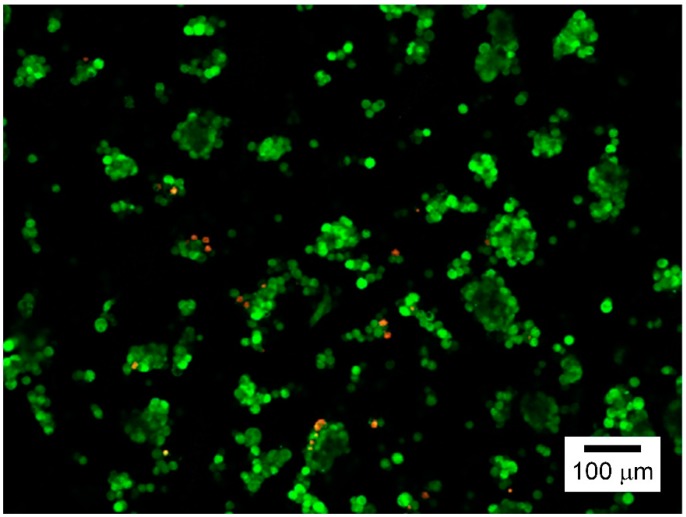
Live and dead cell assay results of the developed microfluidic chip for HepG2 cells (day 1).

The cell viability of a newly built culture system should be determined. Here, we investigated HepG2 cell viability of the microfluidic chip, embracing a nanofiber scaffold via live and dead cell assay. Figure 5 shows the assay results, in which the live cells were stained green, and the red ones corresponded to dead cells. The cell counting assay revealed that the developed microfluidic chip had sufficiently high viability for HepG2 cells (>95%). 

**Figure 6:**
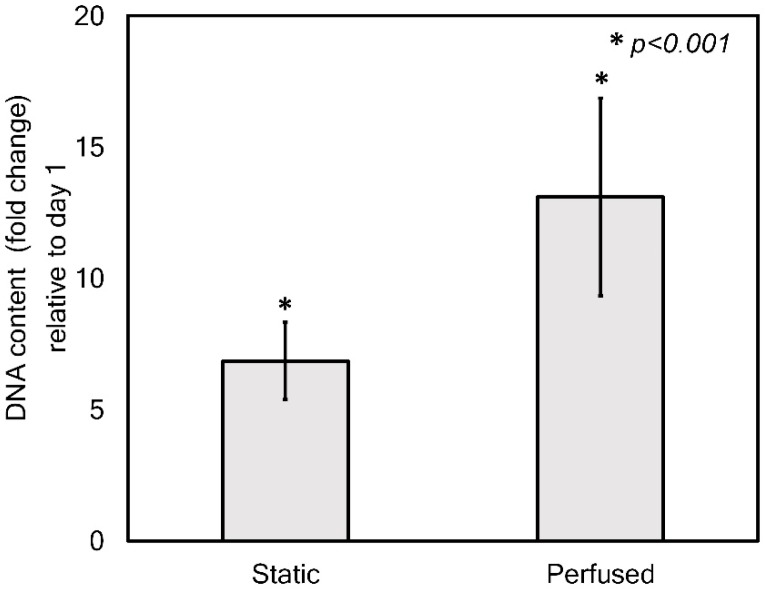
DNA content analysis of the cells cultured under static and perfusion conditions for 1 and 14 days in the chip.

Figure 6 shows the comparison results of the proliferation of HepG2 cells cultured under static and perfused conditions for 1 and 14 days. As shown in Figure 6, the cells cultured for 14 days in the chip successfully proliferated regardless of the medium supply method used, although the standard deviations looked relatively large. The large standard deviation possibly resulted from the variation in cell seeding quality because of the innate uneven fibrous morphology. Even though the cells cultured under static condition revealed excellent proliferation for 14 days (6.6-fold), the perfused condition promoted cell proliferation more. The cells cultured under perfused condition proliferated by more than 12-fold during the same period. Thus, perfusion culture condition in the developed chip gave rise to about two times higher cell proliferation than the static culture condition (*p* < 0.001).

**Figure 7:**
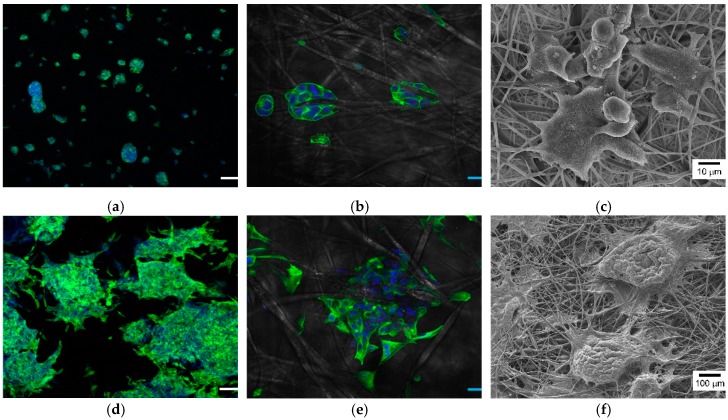
Confocal microscopy and SEM images of the cells cultured on the nanofiber chamber with microfluidic system for 1 (**a**,**b**,**e**) and 14 days (**c**,**d**,**f**). Especially, (**b**,**e**) are enlarged images of (**a**,**d**). The scale bars in the confocal microscopy images are 100 μm (**a**,**d**) and 20 μm (**b**,**e**).

The results demonstrated that the perfusion culture in the developed chip could provide cell-friendly conditions to the cells. To justify the applicability of the developed chip, 3D cell culture and real-time monitoring of the cells’ status using the developed chip were carried out. Figure 7 shows the HepG2 cells cultured for 1 and 14 days in the chip with perfusion. As shown in Figure 7a,b, the cells with the cell length of 16.47 ± 4.84 μm successfully attached and hung on the nanofibers in the form of small spheroids, with diameters ranging from 20 to 50 μm on day 1. Moreover, the cytoskeleton organization, visualized by actin staining, revealed that actin filaments stretched along the nanofibers (Figure 7c). After 14 days, HepG2 cells re-established cell–cell contact and formed about 300 μm-diameter aggregates (or large spheroid) engulfed on nanofibers (Figure 7d–f), which has been reported to be the more desirable formation of HepG2 cells because of higher hepatocyte synthetic functions, higher response for drug toxicity, and better mimicking of in-vivo oxygen gradient in the hepatic lobule [3,24,33]. These results were far from that under 2D culture conditions based on a flat surface. In 2D conditions, HepG2 cells grow as monolayers with flat morphology, which results in short-lived canalicular-like structures [24]. Therefore, the developed microfluidic chip with nanofiber scaffold could successfully provide a 3D cell culture environment. 

**Figure 8:**
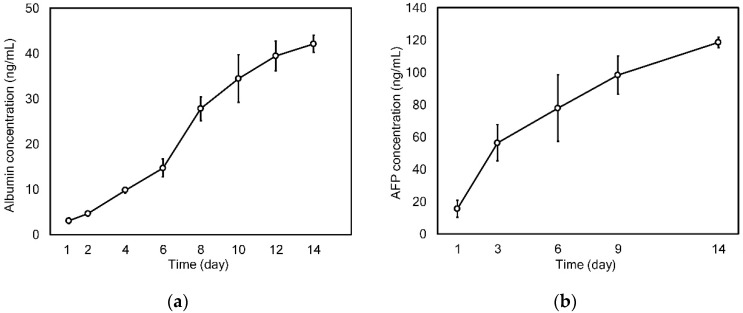
Real-time monitoring of secretion of (**a**) albumin and (**b**) AFP for 14 days.

The typical specific functionality of HepG2 cells was evaluated through real-time monitoring of the concentration of secreted albumin and AFP in the conditioned medium for 14 days. Albumin, which is secreted from HepG2 cells from human hepatocytes, is a marker of metabolic activity of hepatocytes in vitro, and it can indicate liver-specific functions [24]. Thus, the amount of albumin secreted from the cell cultured for 14 days in the chip was examined every two days (Figure 8a). The albumin production rate consistently increased from 3.07 ± 0.61 ng/mL at day 1 to 42.09 ± 1.91 ng/mL at day 14. Moreover, we monitored AFP secretion from the cells because AFP is known as a tumor-associated protein and important indicator of the differentiation of hepatoma cells and disease progression [34]. As shown in Figure 8b, the increase in secreted AFP level was similar to that in albumin secretion for the same duration. The AFP concentration in the conditioned medium collected at day 2 was 15.45 ± 5.13 ng/mL, and this value increased to 118.54 ± 3.19 ng/mL at day 14. As shown in these monitoring results, the developed microfluidic chip embracing nanofiber scaffold enabled the real-time monitoring of cell status based on the conditioned culture medium. 

## 4. Conclusions

In this study, we presented a microfluidic chip with a nanofiber scaffold. Microfluidics can introduce dynamic conditions, and a nanofiber scaffold provides a 3D environment to an in-vitro cell culture. Moreover, the perfusion method in the chip allows the real-time monitoring of cell status with the conditioned culture medium. The microfluidic chip structure was fabricated using well-established soft-lithography at a preliminary level, and a nanofiber scaffold was prepared using electrospinning. In particular, the nanofiber scaffold had various diameter fibers. It was highly porous and heterogeneous in morphology, making it appropriate to provide a human body’s ECM-like 3D environment. To justify the developed chip, HepG2 cells with various protein syntheses and 3D culture-specific morphology were cultured in the chip for 14 days [35]. Results demonstrated that the developed chip had excellent viability (higher than 95%) for HepG2 cells through live and dead cell assay. Additionally, the perfusion-based dynamic culture had a positive effect on the proliferation of HepG2 cells, which was about two-times higher than that under static culture condition. Finally, we presented the application of the chip to 3D cell culture and real-time monitoring based on conditioned culture medium. The cells cultured in the chip successfully formed engulfed aggregates (or large spheroid), which are a 3D culture-specific morphology of the cells. Moreover, albumin levels and AFP secretion from the cells were successfully monitored for 14 days. We expect the concept of the developed chip to be useful for studying 3D cell cultures, live assay or real-time monitoring of cell activity, and in-vitro drug screening and toxicity testing.
